# Simultaneous intraparotid and intratemporal facial nerve schwannoma: resection of the lesion and nerve reconstruction with cross-face technique ‒ Case report

**DOI:** 10.1016/j.bjorl.2026.101761

**Published:** 2026-01-17

**Authors:** José Fernando Polanski, Anelyse Pulner Agulham, Beatriz Alvarez Mattar, Anne Karoline Groth, Marja Cristiane Reksidler

**Affiliations:** a Faculdade Evangélica Mackenzie do Paraná, Curitiba, PR, Brazil; b Universidade Federal do Paraná, Curitiba, PR, Brazil; c Hospital Erasto Gaertner, Departamento de Cirurgia Plástica, Curitiba, PR, Brazil; d Hospital Erasto Gaertner, Departamento de Cirurgia de Cabeça e Pescoço, Curitiba, PR, Brazil

## Introduction

Facial Nerve Schwannoma (FNS) are rare benign indolent growing tumors, derived from Schwann cells.^1^ The tumor may arise anywhere along the course of the facial nerve, and the extra-temporal portion is the most affected.^2^^,^^3^

The real incidence of FNS is difficult to obtain. FNS are more rare than vestibular schwannomas, which have an incidence reported about 20 per million per year. The right and left sides are equally affected. Neither sex exhibits a higher prevalence.^1^

The management depends on the tumor extension, the facial nerve pre-operative function, on the patient's age and preferences. Surgical resection was the ideal treatment until 1995. Nowadays, however, conservative approaches are considered, such as stereotactic radiotherapy or clinical observation with repeated MRI.^2^

In this article, we present a case of facial nerve schwannoma, simultaneously involving the intra-parotid and intra-temporal portions of the nerve. The patient was initially treated with superficial parotidectomy. Later, a closed cavity mastoidectomy on the left, deep parotidectomy, and nerve reconstruction was performed with a microsurgical graft, using the cross-face technique.

## Case report

A 49-year-old female patient sought medical assistance due to a mass in the left parotid region, causing local pain and aesthetic repercussions.

The ultrasound revealed a lesion suggestive of an adenoma in the left parotid gland. A fine needle aspiration biopsy was performed, which was inconclusive. To remove the lesion and confirm the diagnosis, a superficial parotidectomy was performed. During surgery, the intraoperative histopathological analysis was also inconclusive. The anatomopathological examination of the specimen showed a biphasic spindle cell lesion with neural characteristics, compatible with facial nerve schwannoma.

**Fig. 1:**
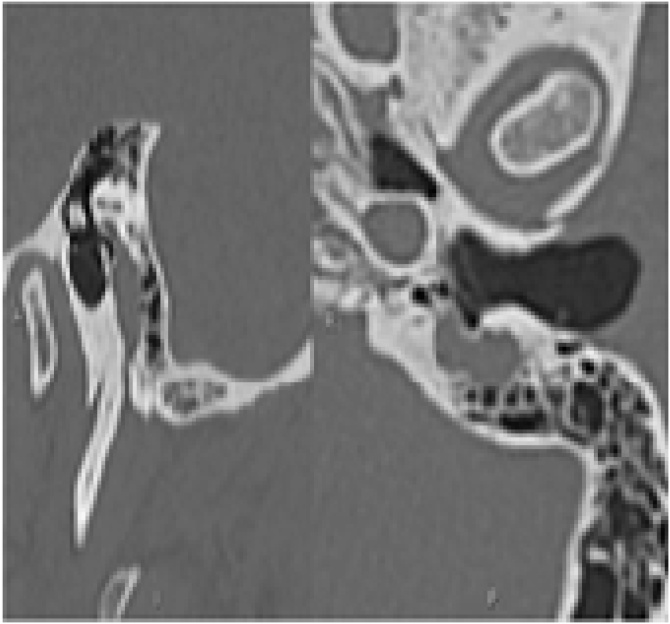
Pre-2nd surgery, temporal bone CT scan (sagittal and axial) showing an expansive lesion in the Fallopian canal, limited to the mastoid portion of the nerve.

After the procedure, the patient had facial palsy, House-Brackmann grade VI (Fisch 0). A new CT revealed a mass in the mastoid region of the left facial nerve canal, expanding from the stylomastoid foramen, which suggested an expansive lesion specific to the facial nerve (Fig. 1). The other portions of the facial nerve were normal.

**Fig. 2:**
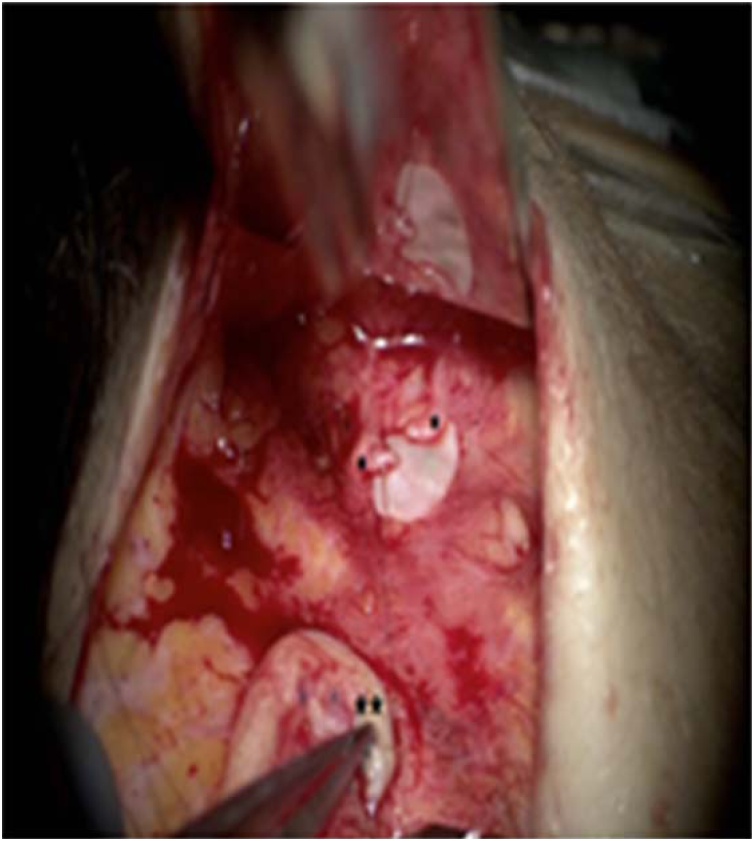
Neurorrhaphy between the facial nerve and the sural nerve graft, using the cross-face technique. ** Sural nerve graft; * Superior and inferior branches of the facial nerve.

**Fig. 3:**
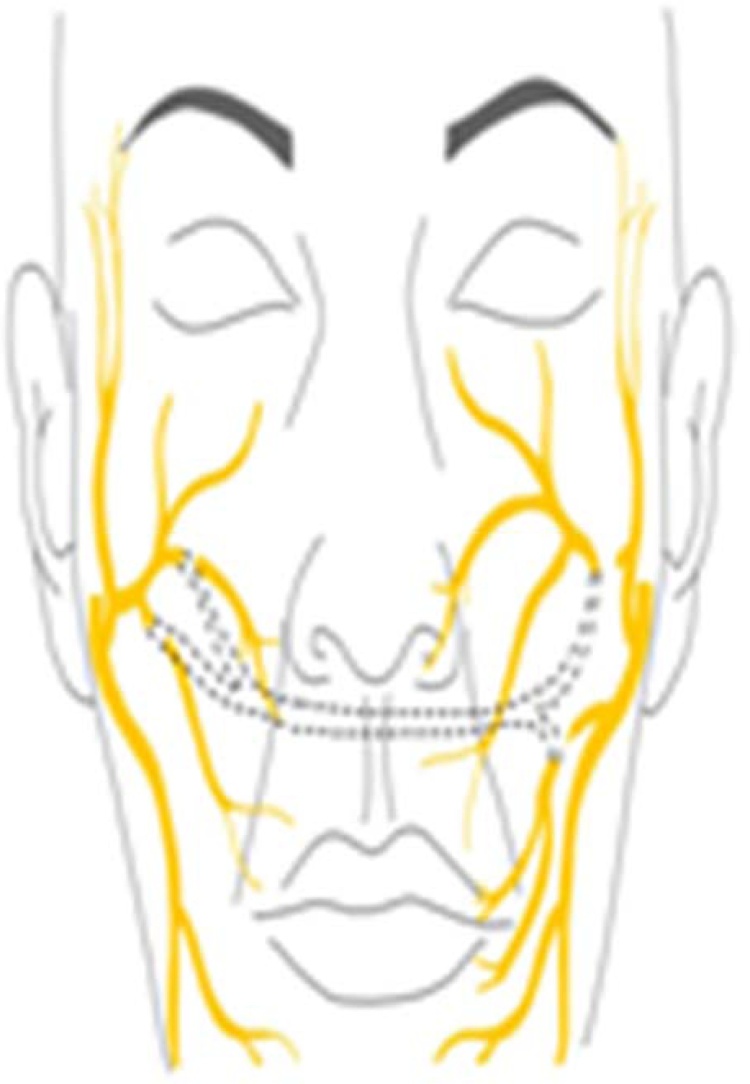
Cross face technique, neurorrhaphy of a nerve graft from the sural nerve with the remaining main terminal branches of the facial nerve on the affected side to the buccal branch of the healthy contralateral nerve.

A new surgical approach was planned for the removal of the lesion and nerve reconstruction. A closed mastoidectomy was performed, as well as facial nerve exploration in the mastoid portion, resection of the tumor along the nerve, and deep parotidectomy. The cross-face technique was used for the nerve reconstruction. A microsurgical graft was used to connect the remaining trunks of the facial nerve on the paralyzed side to the distal branches of the buccal branch on the healthy side, with end-to-end neurorrhaphy (Fig. 2). The graft used was the sural nerve, which was connected to the paralyzed side by a tunnel through the nasolabial region (Fig. 3).

The anatomopathological examination of the mass revealed a schwannoma of the facial nerve.

Facial physiotherapy was recommended post-operatively. One year after the last surgery, the MRI did not show any recurrences. The patient has shown recovery of facial movements, with facial paralysis classified as House-Brackmann IV (Fisch 50).

## Discussion

FNS are rare benign neoplasms, with very few cases reported in the literature.^1^ These tumors, which tend to grow slowly, can develop in any portion of the facial nerve. The most affected site (70%) is the geniculate ganglion, while other portions are rarely affected.^2^ Less than 10% of schwannoma occur in the extratemporal portion of the facial nerve, with intraparotid location being even rarer, representing around 1% of all neurinomas of the facial nerve.^3^^,^^4^

In this case, the patient had facial nerve schwannoma in intratemporal and extratemporal locations, including the parotid gland, and without the involvement of the geniculate ganglion region, as demonstrated by the radiological study, making this report even more unlikely.

The clinical presentation is variable and depends on the location of the nerve affected by the tumor. When there is, as in the case described here, involvement of the parotid gland, as the tissue offers little resistance to expansion, it generally presents as a painless mass, with preserved nerve function. However, if located in the intratemporal part of the facial nerve, symptoms of nerve compression ‒ facial paresis and hemifacial spasms ‒ may occur due to constriction of the bone canal.^2^

Imaging exams have limited diagnostic capacity, especially in parotid masses, but they are capable of demonstrating the extent of the tumor and whether there are simultaneous lesions. Diagnostic confirmation occurs through anatomopathological examination.^1^

If there is more severe nerve dysfunction, facial nerve resection is indicated, whether or not associated with nerve reconstruction.^4^ Some techniques can be used for nerve reconstruction, such as cross-face or interposition nerve grafts on the affected side. Interposition nerve graft with or without sutures, in this case, presented an extra difficulty because the lesion extended along the mastoid region. Accessing the tympanic segment ‒ the portion not affected by the tumor ‒ would involve anatomical challenges in addition to extra risks, such as auditory complications.

Therefore, in this case, reconstruction was chosen using the cross-face technique, which consists of the neurorrhaphy of a nerve graft from the sural nerve that joined the main remaining terminal branches of the facial nerve on the affected side to bundles of the buccal branch of the healthy contralateral nerve.^5^ In this technique, the terminal branches of the buccal bundle are sectioned and sutured to the bundles on the affected side. Electrophysiological monitoring is very useful in locating the appropriate bundles. The buccal branch does not have such a significant impact on facial expression and is therefore generally the best option to be used as an area to be connected to the injured side.

## Conclusion

Facial nerve schwannomas are extremely rare, with very few cases reported in the literature. They present a benign biological behavior and an indolent growth. Despite this, it can lead to facial nerve palsy, affecting patients' quality of life, in addition to the possibility of compression of other anatomical structures. Therefore, an early diagnosis is essential for a more favorable prognosis, with optimized treatment.

## ORCID ID

José Fernando Polanski: 0000-0003-3151-2327

Anelyse Pulner Agulham: 0000-0003-1499-2146

Beatriz Alvarez Mattar: 0000-0001-8603-6060

Anne Karoline Growth: 0000-0001-9888-3099

Marja Cristiane Reksidler: 0000-0001-5974-2074

## Funding

None.

## Data availability

We declare that all data are available in repository.

## Declaration of competing interest

The authors declare no conflicts of interest.
